# Complete Disarticulation of the Upper Maxillary Bones

**Published:** 1869-10

**Authors:** J. H. Salter


### ARTICLE XIII.

### *Complete Disarticulation of the Upper Maxillary Bones.*

By J. H. Salter, M.R.C.S., L.S.A., L.M., A.K.C., etc.

So complicated are the injuries which usually happen to the bones of the upper jaw that no attempt has ever been made, as far as I am aware, to establish a systematic classification of them, or special rules for their treatment. Feeling, therefore, that any addition to the instances already recorded would be acceptable to those who are interested in this subject, I venture to give a short account of a case which recently occurred in my own practice, which to the best of my belief, is unprecedented in the extent of its injury and subsequent result in the annals of Surgery.

In August last, W. S., a labourer, aged 30, was driving a wagon when one of the horses suddenly fell and knocked him down, with his head under the animal. The ground was very hard from the previous drought. When first seen he was sensible, though unable to articulate distinctly; his face was bruised and swelled; his lips and teeth slightly apart, the upper jaw projecting somewhat over the lower, and unable by any effort to be closed upon it. There was no great deformity of the general expression of the face. On touching the cheeks, they appeared to contain a quantity of "loose bones;" on both sides the malar bones were displaced and movable. On laying hold of the upper incisors, the wedge-shape portion of bone corresponding to the position of the superior maxillæ and malars was so movable that the impression conveyed to myself and my assistants was that, by a forcible twist, the whole could have been brought away but for the attachments to the soft parts. At the articulation of the nasal bones with the frontal and lacrymal there was a very distinct separation. The floor of each orbit was depressed and freely movable, the left rather more than the right. The entire jaw seemed to be protruded forward, the teeth being abnormally prominent and overhanging. The alveolar ridges and other portions of the bones were unbroken. The horizontal plates of the palate bones were severed from their connexion with the vertical, and with their articulation with the internal pterygoid processes of the sphenoid, which could be ascertained, on passing the finger along the roof of the mouth, by their extreme mobility. There were no external wounds beyond bruises and abrasion, through œdema and ecchymosis were subsequently considerable.

The appearances above described were clearly made out and recognised by all present, Professional and otherwise, and the disarticulation was beyond a doubt, inasmuch as the bones, in their wedge-shape entirety, could be freely moved backwards, forwards, upwards, downwards, and from side to side. The separation of the malar bones from their articulation was no less distinct. For a considerable time sense of smell was absent, and the tears, by reason of a slight displacement of the puncta, coursed over the cheeks. At no time was there any great pain.

With much time and trouble, I carefully adjusted a gutta-percha casing to the parts. A horizontal slip passed across the upper lip, and exerted backward pressure on the alveolar ridge, to obviate its tendency to eversion. This was joined by two lateral flaps brought from the top of the head (corresponding with the coronal suture) beside the cheeks, and united with another horizontal slip passing from the back of the head below the occiput to either side, to steady and keep in position the two malar bones. These were carefully padded with strips of spongio-piline, which readily adhered to the gutta-percha when hot. Over all, a bandage was put, fixing firmly the lower jaw on the upper by exerting upward pressure. He was fed through an opening of his teeth with fluid food.

In the course of five or six weeks I removed the gutta-percha apparatus, and put on a starch bandage for another fortnight. It was several months before he could bite solid food. He is now quite convalescent, and very little the worse for his accident, though, as if to bear testimony to the curious nature of the injury, the upper jaw appears to be set slightly askew, and the depressions between it and its articulations are abnormally wide.—*London Med. Times & Gazette.*

